# A survey and optical microscopy in pilot comparative analysis of generic and original nimesulide granules

**DOI:** 10.1016/j.heliyon.2021.e07490

**Published:** 2021-07-06

**Authors:** Pavlo Leonenko, Natalia Ostanina, Yuliia Kokoieva, Mykhaylo Levin, Halyna Leonenko, Oleksii Gumeniuk, Olena Doroshenko, Yana Nikolaieva

**Affiliations:** a Institute of Dentistry, Shupyk National Healthcare University of Ukraine, Kyiv, 04112, Ukraine; b State Institution “O.M. Marzieiev Institute for Public Health” of the National Academy of Medical Sciences of Ukraine, Kyiv, 02094, Ukraine; c Department of Orthodontics and Prosthodontics Propaedeutics, Bogomolets National Medical University, Kyiv, 01601, Ukraine

**Keywords:** Nimesulide, Generic drugs, Particle size, NSAIDs

## Abstract

**Background:**

Secondary to increased development of generic nonsteroidal anti-inflammatory drugs (NSAIDs), there is a lack of simple and inexpensive ways of pilot detection of differences between the batches of generic drugs and the original ones.

**Objectives:**

To determine the peculiarities of the use of generic NSAIDs in routine practice through a pilot survey of dentists and to conduct a pilot comparative analysis of generic and original NSAIDs containing nimesulide granules using optical microscopy.

**Methods:**

The first part of the study included a pilot survey Convenience sampling of 192 dentists to study the use of generic NSAIDs in their routine practice. The second part included the use of a pilot optical microscopy of nimesulide particles isolated from four drugs: original drug (NA) and generic ones (NB, NC, ND).

**Results:**

In the questionnaires, dentists pointed to a 68.7% lower clinical efficacy and a 62.6% higher percentage of side effects of generic NSAIDs compared to the original ones. Based on the results of pilot optical microscopy, a statically significant difference in the size distribution of the drug substance particles in all generic nimesulide granules was determined as follows: NB (χ^2^: 15.15; p < 0.01); NC (χ^2^: 11.09; p < 0.05); ND (χ^2^: 1625.34; p < 0.001) compared with the original drug NA.

**Conclusions:**

A pilot survey of dentists showed that doctors noted the practical difference in the effects of the original and generic NSAIDs. A significant difference in the size of nimesulide particles and their distribution in generic drugs NB, NC, ND compared to the original NA suggests a possible difference in bioavailability and bioequivalence.

## 1 Introduction

Drugs are divided into two categories according to the innovation criteria: 1 – original; 2 – generic (reproduced) [1]. Original drugs are drugs that went through the entire cycle of preclinical and clinical trials; the original formula of their active pharmaceutical ingredient (API) is patented [2]. Generic drugs are drugs with proven pharmaceutical, biological and therapeutic equivalence to the original drug [1].

According to the definition of the U.S. Food and Drug Administration (US-FDA), a generic drug matches the original drug in terms of the dose of the active pharmaceutical ingredient, the magnitude of effect, route of administration, quality, indications to use, and appearance and can be used interchangeably with the original drug [2].

The development of an original drug is a long and costly process that involves many highly qualified specialists; it may last 10–15 years and require multi-million investment [1]. Taking into account the in-depth data from the comprehensive study of the original drug and high material liability, it may be assumed that all series of the original drug that are manufactured and marketed are therapeutically equivalent to the series, for which the pre-clinical and clinical trials were performed and the therapeutic and toxicological profiles were established.

In order to develop a generic drug, a minimum set of studies both for the API and the drug need to be conducted. Although the manufacture of both generic and original drugs must comply with the Good Manufacturing Practice (GMP), which controls the compliance of the drug manufacture process with international pharmacological protocols in order to ensure pharmaceutical, biological and therapeutic equivalence to the original, it is often ignored by the manufacturers of generic drugs. The difference of generic drugs also consists in the financial expenses for the development as, in order to make drugs cheaper, manufacturers sometimes don't conduct clinical trials or comparative trials with the original, neglect the study of drug safety or market the drug without patent protection [1]. This approach decreases the cost of generic drug development. As a consequence, a large number of new generic drugs appear in the pharmacological market.

Nowadays, the pharmacological market is progressing rapidly. The dynamics indicates powerful development of the industry of generic drugs as such that are more affordable, competitive and attractive for users as compared to original drugs [3, 4]. However, taking into consideration the incomplete knowledge and low material liability for generic drugs, some drug series on the market may differ considerably from the bioseries which was studied and for which biological equivalence with the concrete series of the original drug was established. The difference in the composition and structure of the drug (physical and chemical properties of particles, related substances) and the composition of excipients cause lack of drug bioequivalence. Hence, the loss of bioequivalence with the original drug and, accordingly, of therapeutic equivalence of the generic drug brings about insufficient pharmacological activity, clinical effect and safety profile [5].

In order to provide the population of Ukraine with high-quality medicinal products, it is necessary to use a simple, inexpensive, not requiring unique equipment and rapid method of pilot discriminatory analysis and detection of anomalous lots of Nimesulide granules for oral suspension at the market. To solve this problem, according to the literature, the following approaches and techniques that are complex in performing and expensive are most often used. To determine whether generic drug is bioequivalent to the original drug, the Biopharmaceuticals Classification System (BCS) is used, approved by the US-FDA in 2000 and the European Medicines Agency (EMA) in 2001 and afterward by the World Health Organization (WHO), the International Pharmaceutical Federation (FIP) and regulatory authorities around the world [6]. The procedure for using BCS is called biowaiver and refers to comparative *in vitro* studies [7]. Based on degree and rate of absorption which depend on dissolution, aqueous solubility and intestinal permeability, drugs are divided into 4 classes [8]. Nimesulide belongs to the II class of BCS. It is a sparingly soluble compound, therefore is characterized by high permeability through the membranes of the gastrointestinal tract (GIT) and low solubility [9].

However, despite the fact that the biowaver method is being actively developed and improved, and accelerate the procedure of determination the drug's bioavailability without *in vivo* studies [10], for class II drugs the use of this method alone is not enough informative and requires additional studies.

Besides biowaiver method, one of the methods for comparing bioavailability is the Dissolution test. It is common accepted in the Pharmacopoeia of many countries and allows you to determinate the bioavailability of the drug *in vivo* [11, 12]. However, the Dissolution test requires long training, highly qualified personnel, expensive equipment and significant time costs [9, 13]. In addition, the use of this technique for BCS class II drugs is complicated because there are difficulties in choosing the medium for dissolution [13]. Inconsistency in rules and criteria between different countries, differences in the interpretation of test results for assessing the similarity of dissolution profiles is another key point that complicates this procedure and does not allow its use for rapid pilot identification of anomalous lots of generic nimesulide drugs [14, 15].

Till date, only few works have been published in the available literature on methods of pilot discriminant analysis of generic nimesulide granules for oral suspension, which would allow rapidly and without the use of equipment for hundreds of thousands of dollars to detect differences between lots of generic and original drugs and send suspected or anomalous lots for additional complex abovementioned studies.

The aim of the study is to determine the peculiarities of the use of generic non-steroidal anti-inflammatory drugs (NSAIDs) in dental practice by pilot survey of dentists, to clarify satisfaction with outcomes of their patient after prescribing generic NSAIDs, and to conduct a pilot comparative analysis of generic and original NSAIDs containing nimesulide granules using optical microscopy.

## 2 Material and methods

To achieve the aim, this study was divided into two parts. In the first part, a pilot survey of practicing dentists who came from different regions of Ukraine for the annual planned postgraduate studies at the university where investigators work. One of the tasks of the pilot survey was to determine the peculiarities of generic NSAIDs use in dental practice, as well as to analyze the satisfaction of dentists with the results of prescribing generic NSAIDs to their patients. In addition, it was necessary to determine which dosage forms of NSAIDs are most often used by dentists, as well as to determine which of the four commercially available brands of nimesulide granules for oral suspension respondents prefer - original or generic.

In the second part of the study, we planned an attempt to explain the results obtained in the first part by identifying significant physicochemical differences between the brands of nimesulide granules for oral suspension mentioned in the questionnaires for dentists. According to investigators, the factor that causes these differences is the difference in particle size distribution of the active pharmaceutical ingredient, nimesulide. Quantitative optical microscopy is planned to be used for pilot tests and analysis of this factor.

The study design was approved by the Ethics Committee of the Shupyk National Healthcare University of Ukraine and meets the principles of bioethics and legal norms and requirements for trials.

### 2.1 The first part of the study: survey of dentists

**Figure 1:**
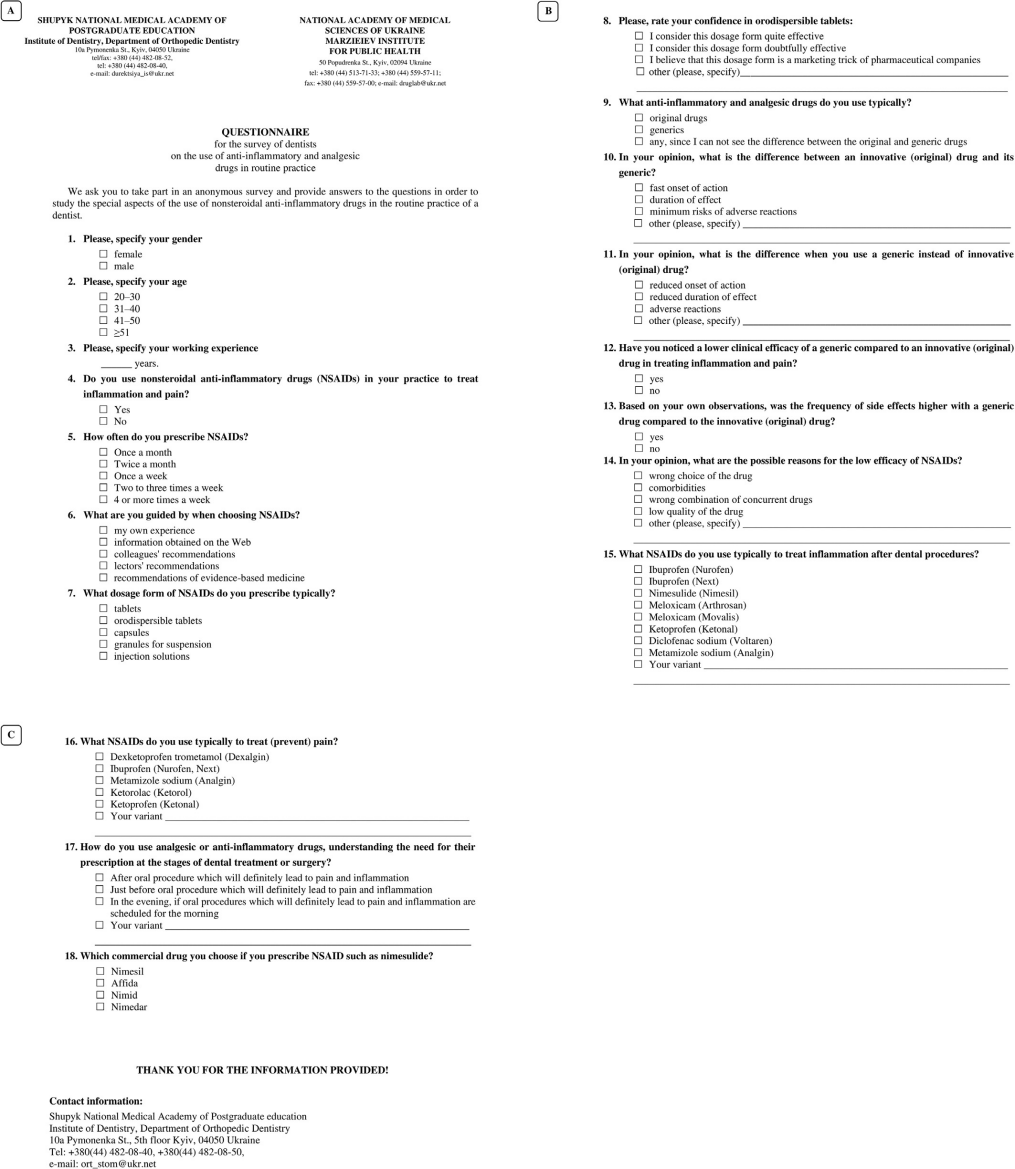
Developed by investigators Questionnaire for the survey of dentists on the use of anti-inflammatory and analgesic drugs in routine practice: (A) page 1, (B) page 2, (C) page 3.

The 18-point questionnaire was used for an anonymous survey of practicing dentists from different regions of Ukraine. The questionnaire was developed by investigators and consisted of questions of various formats: demographic and gender questions, multiple choice question and open-ended questions (Figure 1). The survey can be conditionally divided in groups by question objectives. The first group provides understanding about the gender identity and the professional level of respondents and about his general vision as to the administration of NSAIDs. The second group of questions provides understanding about the success and issues related to the use of NSAIDs in dentistry and about the clinical effectiveness of the use of generic drugs and their commercially available names. The third group of questions provides answers to questions, which commercially available NSAIDs the respondents prefer and why.

Sampling Method. To achieve the aim, in the first part of the study a pilot anonymous survey of a convenience sampling of practicing dentists has been conducted at the Shupyk National Healthcare University of Ukraine in the period from January 2019 to January 2020 [16].

Criteria for inclusion of dentists into the study. The study invited dentists who came to postgraduate continuous medical education. After receiving comprehensive information on the study, 192 individuals voluntarily participated in an anonymous pilot survey.

Exclusion criteria from the study. Dentists who refused to participate were not included in the study and did not receive the questionnaires. Dentists who do not prescribe NSAIDs in their clinical practice did not receive the questionnaires. A total of 192 questionnaires were distributed. Questionnaires that did not answer at least one question were excluded from further study.

After confirming, by way of surveys, the relevance of the problem of choosing NSAIDs for the prevention of inflammation or pain and the need to ensure maximum clinical effectiveness and to minimize the risk of complications due to the administration of generic nimesulides, as well as understanding the commercial names of generic nimesulide granules used by dentists, a pilot comparative analysis using optical microscopy was planned.

### 2.2 The second part of the study: quantitative optical microscopy for pilot detection of differences in the distribution of particles of the active pharmaceutical ingredient by size in commercially available generic and original nimesulide granules for oral suspension

For the second part of the study, a series of direct tests of the particle size distribution of the active pharmaceutical ingredient in drug solid (granules for oral suspension) of original and generic nimesulide drugs of four different manufacturers were conducted at the State Research and Development Laboratory of Drug Quality Control of the State Enterprise “O.M. Marzieiev Public Health Institute” of the National Academy of Medical Sciences of Ukraine within the state-funded scientific program “Development of the methodology for choosing generic drugs to provide the Ukrainian population with high-quality drugs”, topic code АМН.14.18. For the purposes of the study, the following nimesulide drugs were bought at a pharmacy chain: the original drug that had lost patent protection – NA and three of its generic, commercially available analogue drugs: NB, NC, ND (Table 1).

**Table 1:** Nimesulide granules for oral suspension selected for the study.

Drug code in the study	Drug type	Brand name	Manufacturer
NA	original	Nimesil	Berlin-Chemie/A.Menarini, Italy
NB	generic	Affida	Schonen, Switzerland
ND	generic	Nimid	Kusum Pharm Ltd., Ukraine
NC	generic	Nimedar	PRJSC Pharmaceutical Company Darnitsa, Ukraine

Our proposed method of pilot comparative analysis of commercially available nimesulide granules was based on finding a difference in particle size distribution of the active pharmaceutical ingredient nimesulide in three generic (NB, NC, ND) and original (NA) drugs. Pharmacopoeial quantitative optical microscopy was chosen as the final analytical operation of this procedure. However, since the granules of NB, NC, ND and NA contain only 100 mg of the active pharmaceutical ingredient nimesulide and approximately 1900 mg of excipients, a number of preparatory stages had to be performed to conduct the final analytical operation. The first stage consisted of obtaining a dry active pharmaceutical ingredient and a solid phase dispersion of the drug, followed by quantification of nimesulide. After isolation of the drug and determination of the weight of obtained nimesulide dry residue for each of studied drugs, the next stage of the study was performed - the stage of identification of the drug substance and its validation. The final analytical operation was performed at the last stage of the study using the pharmacopoeial method of quantitative optical microscopy.

#### 2.2.1 The stage of receiving solid API and solid-phase API dispersion, nimesulide assay

At this stage, the contents of five sachets (total weight 10,000 mg) with each of the drugs (one sachets weight is 2,000 mg) was put in a 500 ml cylindrical container, 500 ml of water was added, the container was closed, shaken intensively for 5 min and left in a dark place for 72 h. The supernatant was then decanted to the residual volume of 10 ml. 500 ml of water was added to the resulting residue, the container was closed, shaken intensively for 5 min and left in a dark place for 72 h. Given the low solubility of nimesulide 5.5 μg/ml at room temperature 25 °C [17], as a result of its dissolution in two portions of water of 500 ml (total 1000 ml of water) was expected to obtain a saturated solution (that means the substance concentration in solution is equal to its solubility) with only 5.5 mg (1000 ∗ 5.5 = 5500 μg) of nimesulide, which will be 1.1% of the nominal content of nimesulide in 5 sachets (5 ∗ 100 = 500 mg). Given the extremely low solubility of nimesulide and the high solubility of excipients (macrogol cetostearyl ether, sucrose, maltodextrin, citric acid anhydrous and orange flavor) [17, 18, 19] after two processing of the contents of five single-dose sachets of each studied drug with 500 ml water, nimesulide API should remain in the sediment, and excipients after their dissolution should go into the supernatant solution and be discarded during the decantation of the supernatant. Based on the above, to complete the sample preparation after the second addition of 500 ml water and waiting 72 h, the supernatant was then decanted to the residual volume of 10 ml, the liquid with the nimesulide API residue was transferred to a small vial and allowed to stand until the nimesulide API residue was markedly separated from the almost transparent water, which was then decanted.

The nimesulide API residue in the vial was dried in a vacuum drying cabinet at a temperature of 60°С and a residual pressure of 0.1 bar for 2 h. The mass of the nimesulide API dry residue was then determined for each of the four studied nimesulide granules drugs.

The completeness of the excipient separation from nimesulide was monitored in the next stage of the study during drug substance identification and validation.

#### 2.2.2 Identification of active pharmaceutical ingredient and its validation

For API identification and validation, after the foregoing procedure of sample preparation, from the dry residues of each of four drugs was taken 2 mg of nimesulide API, mixed with 600 mg of fine powder of potassium bromide (1:300). A disk was formed from part of this mixture and the infrared spectrum of each of the dry drug residues and the spectrum of the pharmacopoeial reference standard of nimesulide were recorded using an IR spectrometer (IRAffinity-1, SHIMADZU, Germany) [20]. The standard match ratios were calculated in percentage and compared to the library standard.

After the sample processing of the solid dispersion of the active pharmaceutical ingredient with the preservation of large particles and the IR-spectrometry for the determination of the identity of IR-spectra of studied drugs, microscopy samples were prepared. The procedure of preparing a sample was as follows: a 1.0 mm wide 25.4 х 76.2 mm slide was put on the analytical balance, the slide was calibrated, then the nimesulide API dry residue with the target weight 5mg was introduced by spatula and immersion oil (Microscopy Immersion oil, Merck, Germany) with the target weigh 70 mg was added. After recording the actual weight of the nimesulide API dry residue and immersion oil, the obtained data were entered into the table.

#### 2.2.3 Quantitative optical microscopy stage

After recording the weight of API dry residue and the immersion oil, under a microscope with the help of a fine needle, the dry residue and the immersion oil were placed on the glass slide and thoroughly mixed so as to destroy the druses of nimesulide particles and prepare the API for counting the particles. On this glass slide with nimesulide API and immersion oil, a second slide then was placed and under its weight the immersion oil and nimesulide API particles were distributed over most of the area between the two slides.

Quantitative optical microscopy as the final analytical operation was performed using an optical digital microscope (MBL 2000, A.KRÜSS Optronic, Germany) with a digital camera (Delta Optical DLT-Cam Basic, Delta Optical, Poland) with a 1600 х 1200 pixel resolution [21, 22, 23, 24]. During the optical microscopy, photographs were made and saved in the jpeg format for further processing in the CAD/CAE software (Autocad, Autodesk, USA) with the size of the photographed area being 620 х 465 μm. The entire area of the sample was analyzed for the presence of particles larger than 30 μm. Given that for final analytical operation we used the pharmacopoeial method of quantitative optical microscopy at all stages of its implementation, including the stage of processing the obtained images of API particles in the CAD/CAE software, we determined the shape, number of particles and conditional dimensions of the particles using the length of the diagonal (D) of the rectangle and sides L1 and L2 according to formula (1):

(1) $$D=\sqrt{L_{1}^{2}+L_{2}^{2}}$$

based on the general article of the pharmacopoeia [21, 22], using the guidelines of the monograph [23], and placement on the resource [24]. These data were exported to electronic tables and further compared in terms of size and differences in the number of particles for various size ranges of nimesulide particles in the generic and original drugs [21].

The obtained date was exported to the Statistica v.12.6 software (StatSоft, Inc, USA). The survey results were exported into tables and analyzed using descriptive statistical methods. The results of quantitative optical microscopy were investigated for the shape of data distribution using the Kolmogorov - Smirnov test. Since the data distribution was not normal, the statistical analysis was conducted using the χ^2^ test to compare the distribution API particle sizes for the generic and original drugs. The value of p ≤ 0.05 was considered statistically significant.

## 3 Results

### 3.1 Results of the first part of the study: survey of dentists

Received after filling 192 questionnaires by dentists. Of the 192 questionnaires, 147 contained comprehensive answers to all questions of questionnaire (response rate 76.6%). 45 dentists’ questionnaires were partially completed (had missed answers to one or more questions) and were excluded from the study.

Of the 147 dentists who completed the questionnaire, 76 women (51.7%) and 71 men (48.3%), with an average work experience of 8 years. According to the survey of 147 dentists, NSAIDs are used by 94.6% (n = 139) of the respondents, of which 41.5% (n = 61) respondents prescribe NSAIDs 2–3 times a week, 32.7% (n = 48) prescribe them once a week, 14.3% (n = 21) 4 and more times a week, 6.8% (n = 10) twice a month, and 4.8% (n = 7) once a month. Dentists are mostly guided by the recommendation of evidence-based medicine when choosing NSAIDs – 54.4% (n = 80) of the respondents. However, when choosing NSAIDS a significant number of dentists are guided by other sources: 23.1% (n = 34) are guided by lectors' recommendations, 12.2% (n = 18) by their own experience, 10.2% (n = 15) by colleagues’ recommendations. According to the survey, the most frequent dosage form of NSAIDs prescribed by the dentists are granules for suspension – 48.3% (n = 71).

According to the survey, 71.4% (n = 105) of dentists use original drugs, whereas 11.6 % (n = 17) do not see any difference between the original and generic drug, and 17.0% (n = 25) of respondents prescribe generic drugs. 68.7% (n = 101) of dentists who took part in the survey noticed lower clinical effectiveness of generic drugs as compared to original ones in combatting inflammation and pain. Besides, 62.6% (n = 92) of respondents noted a higher percentage of side effects when prescribing generic drugs as compared to original ones.

In order to manage inflammation following dental interventions, dentists-respondents most frequently used such NSAIDs as nimesulide 67.3% (n = 99) and ibuprofen 23.8% (n = 35). Other respondents prescribe various combinations of the following NSAIDs: Meloxicam, Ketoprofen, Diclofenac sodium, Metamizole sodium. When choosing a commercially available nimesulide drug, 74.1% (n = 109) of respondents preferred the original Nimesil. At the same time, 25.9% (n = 38) of respondents preferred generic commercially available nimesulide drugs, such as Affida, Nimidar and Nimid.

Thus, taking into account the survey results, the following NSAIDs were selected for the second stage of laboratory tests: original Nimesil and all commercially available generic nimesulide drugs listed by the respondents in the survey: Affida, Nimedar and Nimid.

### 3.2 Results of the second part of the study: quantitative optical microscopy for pilot detection of differences in the distribution of particles of the active pharmaceutical ingredient in commercially available generic and original nimesulide granules for oral suspension

#### 3.2.1 Obtaining dry API and solid-phase dispersion of the API, nimesulide assay

When preparing samples of four studied drugs for microscopy, immersion oil was added to the dry residue. Actual weights were recorded for each of the samples (Table 2).

**Table 2:** Actual weights of components for the microscopy of studied drugs.

Sample component	Weights of sample components for microscopy, mg			
	NA	NB	ND	NC
Dry residue of nimesulide API	4.6	4.5	5.0	5.1
Immersion oil	70.5	65.0	65.1	71.1

The actual weight of the investigated dry residue for each of the products averaged 4.8 mg. The actual weight of immersion oil averaged 67.7 mg.

#### 3.2.2 Results of the API identification and validation stage

**Figure 2:**
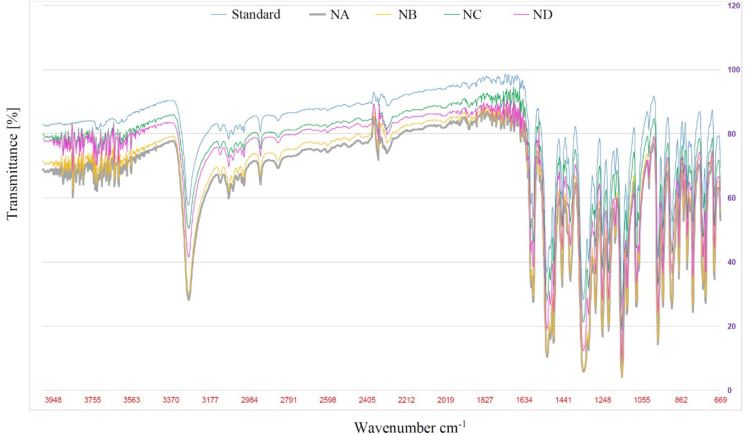
Infrared spectra of dry residues API of studied drugs and the pharmacopeial nimesulide standard sample, (standard) IR spectrum of the pharmacopoeial reference standard of nimesulide, (NA) IR spectrum of the investigated API of the original drug nimesulide granules, (NB, NC, ND) IR spectra of the investigated API of three generic drugs of nimesulide granules.

After sample processing and IR spectrometry of each of four dry residues of the studied drugs and the pharmacopeial standard sample of nimesulide, we analyzed the data and obtained the following standard match ratios: 90.5% for NA, 90.6% for NB, 89.4% for ND, and 89.7% for NC. As a result of IR-spectrometry, the obtained spectra were almost identical (Figure 2). Validation results confirm that dry residues of all samples contained nimesulide API. Thus, as a result of sample processing pure nimesulide API samples were obtained with the preservation of large particle fractions, which is necessary for the next stage of laboratory tests.

#### 3.2.3 Results of quantitative optical microscopy

**Figure 3:**
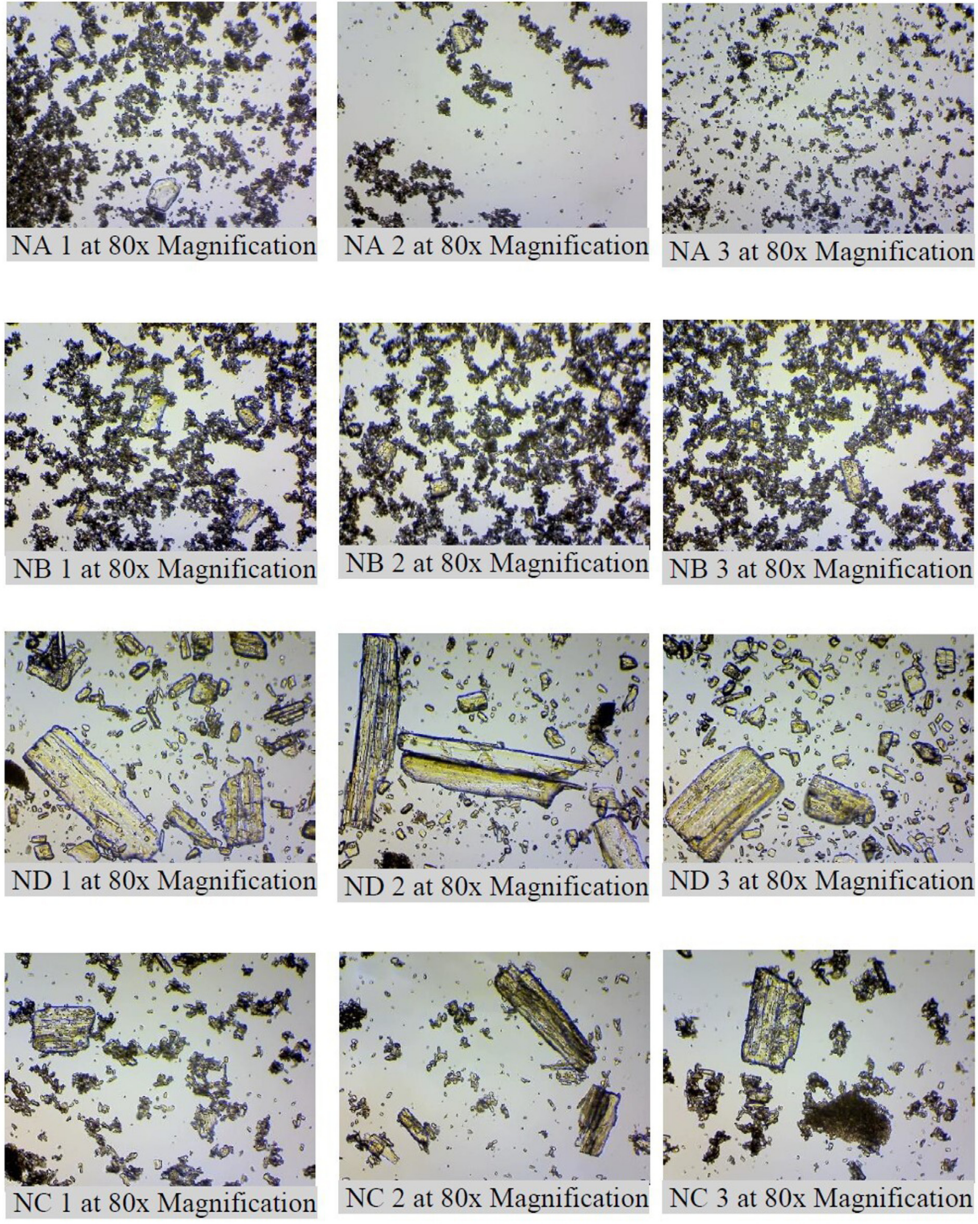
Typical photos of the studied drugs NA, NB, NC, ND taken during optical microscopy of nimesulide API particles with a magnification of 80x, (NA 1-3) particles of the investigated original nimesulide API granules, (NB, ND, NC 1-3) particles of three investigated commercially available generic nimesulide API granules.

During the optical microscopy using a digital microscope and a digital camera, photo records of the study were collected, and the photographs shown in Figure 3 were imported for further measurements. The typical photos provide evidence of significant differences between the particles of generic drugs NB, NC and ND and the original NA drug in terms of shape, size and quantity distribution.

During the processing of photos of the studied drugs in the CAD/CAE software, particle size and quantity measurements were imported to a research database and summarized in Table 3 after statistical processing. During the statistical analysis, the percentage of particle quantity for each size range was determined in proportion to the total number of particles in each nimesulide drug (Table 4).

**Table 3:** Distribution of nimesulide API particles in various size ranges in investigated NA, NB, NC, ND nimesulide API granules.

API particle size range, μm	Number of API particles, units			
	NA	NB	NC	ND
30–40	601	2838	4128	182
41–50	264	637	2501	799
51–60	135	271	1495	1795
61–80	43	79	1152	3274
81–120	-	13	505	2402
121–160	-	-	33	479
161–200	-	-	17	155
201–240	-	-	3	59
241–400	-	-	-	46
Total number of API particles	1043	3838	9834	9191

**Table 4:** Percentage of particles in the nimesulide API in investigated NA, NB, NC, ND nimesulide API granules.

API particle size range, μm	Percentage of the amount of API particles, %			
	NA	NB	NC	ND
30–40	57.62	73.94	41.98	1.98
41–50	25.31	16.60	25.43	8.69
51–60	12.94	7.06	15.20	19.53
61–80	4.12	2.06	11.71	35.62
81–120	-	0.34	5.14	26.13
121–160	-	-	0.34	5.21
161–200	-	-	0.17	1.69
201–240	-	-	0.03	0.64
241–400	-	-	-	0.50

**Figure 4:**
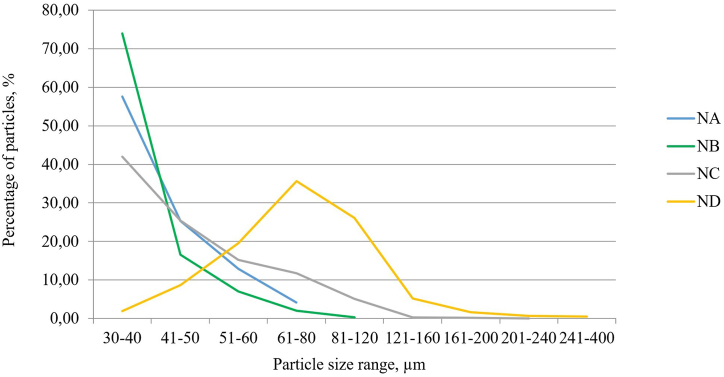
The frequency of drug particles in the four studied drugs (NA, NB, NC, ND) as a percentage in each of the presented ranges of particle sizes of nimesulide.

The distribution of data was not normal (Figure 4). The χ^2^ test demonstrated a statistically significant difference (р<0.05) in the distribution of particles in all generic drugs (NB, NC, ND) as compared to the original one (NA). The results of the χ^2^ test are provided in Table 5. The closest distribution in terms of the particle size was found in NA and NC (χ^2^ = 11.09; р<0.05), but NC differed in the presence of large particles. A significant difference was found for ND as compared to NA (χ^2^ = 1625.34; р<0.001).

**Table 5:** Results of χ^2^ determination in pairs of comparisons of particles distribution groups in investigated drugs (generic API (NB, NC, ND) and original API (NA)).

Comparison groups	χ^2^ value	Р value
NA and NB	15.15	<0.01∗
NA and NC	11.09	<0.05∗
NA and ND	1625.34	<0.001∗
NB and NC	44.21	<0.001∗
NB and ND	2687.58	<0.001∗
NC and ND	880.47	<0.001∗

∗ Differences are statistically significant.

## 4 Discussion

Dental interventions often involve pathological processes such as inflammation and pain. Prevention and management of these processes is an important objective in dentistry. According to literature, a scientifically justified drug for controlling pain and inflammation is nimesulide, which is due to its high clinical effectiveness, safety profile and short onset of action [25, 26, 27, 28]. According to the results of our survey, dentists most frequently use nimesulide to manage inflammation following dental interventions. That is why the original nimesulide drug and its generic counterparts were selected for further laboratory tests.

According to the scientific literature, the development of generic drugs on the pharmacological market currently surpasses that of original drugs owing to smaller financial and time expenses [29]. However, generic drugs do not always demonstrate sufficient clinical effectiveness. It is often associated with a different API structure and, as a consequence, lack of bioequivalence between the generic and the original drug [5].

At the same time, different investigators noted not rare losses of therapeutic equivalence of generic NSAIDs, reduction of their pharmacological activity, modification of the clinical effect and changes in the safety profile. All this leads to a loss of confidence in generic drugs in general. According to the results of systematic review of 52 articles, which aimed to investigate negative perceptions about generic medicines and evaluate the proportions of lay people, doctors and pharmacists who hold these perceptions, a negative attitude towards generic drugs was confirmed [30].

Based on doubts about the effectiveness of generic drugs in 35.6% of lay people and the fact that 33.4% of pharmacists considered generics of lower quality than the original drugs, and 24.4% of doctors believed that generics cause more side effects than original drugs, the authors of this systematic review concluded that a significant proportion of physicians, pharmacists, and nonprofessionals are negative about generic drugs [30].

Taking into account the not rare results of scientific studies that prove the lower effectiveness of generic drugs, we planned and conducted a pilot survey of dentists to determine the peculiarities of using the NSAIDs in their dental practice. One of tasks was to clarify the satisfaction of dentists with the results of prescribed generic NSAIDs to their patients, including nimesulide granules (the leader of prescriptions by dentists in Ukraine in 2017, 2018) [31]. Discussion of the results of the pilot survey of dentists considers that we used such a sampling method as Convenience sampling, which does not allow scientific generalization of the results of the survey of all enrolled respondents to the entire population of dentists in Ukraine. Accordingly, this limitation was taken into account in further discussions and conclusions. Summarizing the obtained survey results, the following key findings should be noted. Firstly, most of the respondents are guided by the evidence-based medical data when prescribing NSAIDs, but over 45.6% (n = 67) of dentists still rely on their experience or on the opinion of more experienced specialist when making prescriptions. This approach may lead to adverse consequences and requires explanatory work among specialists concerning the advantages of the scientifically grounded prescription of any drugs, including NSAIDs. This can be done through scientific publications, specialized associations and continuous post-graduate education. Secondly, regardless of the fact that various NSAIDs, both original and generic, are available on the Ukrainian market, according to the survey, respondents prefer NSAIDs with nimesulide as the active ingredient in the dental anti-inflammatory therapy, which is in line with the findings of other studies [31, 32]. Among commercially available nimesulide drugs, dentists definitely prefer the original NA drug, which also complies with other scientists’ findings [31, 32], and not only in stomatology but also in general medical practice [33]. Thirdly, dentists-respondents do not trust generic NSAIDs pointing to a number of reasons for distrust in their surveys: low clinical efficacy reduced onset of action, reduced duration of effect and a high percentage of side effects. It is a known fact that the bioavailability of a drug containing API in solid form (examples of such drugs are: tablets, capsules, suspensions for oral administration, suspensions for parenteral administration, granules for the preparation of suspensions) depends largely on the particle size distribution of drug substance [21].

Therefore, based on this fact and the fact that majority of dentists-respondents in the questionnaires noted lower clinical efficacy of generic NSAIDs and a higher percentage of side effects of generic drugs compared to the original, we tried to explain these results by identifying differences in the distribution of API particle size distribution in granules of nimesulide. To solve this problem ad hoc, we used pharmacopoeial quantitative optical microscopy, based on the fact that knowledge of the physicochemical properties of API makes it possible to predict the rate of dissolution and absorption of API [9].

Additionally, based on the crystallography data, a difference in the API particles distribution in the drug leads to changes of the dissolution rate and, as a consequence, changes of drug bioavailability since there is a correlation between the dissolution rate and the API particle size. Spectral analysis demonstrated that all studied drugs complied with the pharmacopeial standard nimesulide sample. Based on this, NB, NC and ND generic drugs must be identical to the NA original drug both in terms of particle size and shape and in terms of particle distribution in the API. That is why in our study the original NA drug was taken as a standard of particle distribution. As a result of the laboratory tests and statistical processing, a statistically significant difference in particle distribution was established for all generic drugs: NB (χ^2^ – 15.15, р<0.01); NC (χ^2^ – 11.09, р<0.05); ND (χ^2^ – 1625.34, р<0.001) as compared to the original NA drug.

As is known from research, any differences in the API structure between the generic and the original drug affect the drug solubility and absorption and cause inadequate clinical effect and complications. According to researchers [34], the particle distribution by size significantly influences the drug solubility and, accordingly, its bioavailability. In the above study, four commercially available nimesulide drugs were studied: RA, TS, TB and TC. Based on the dissolution test, full dissolution of RA and TS drugs was recorded; their particles were identical in size. Only 80% of the TB drug and the least of the TC drug were dissolved; these drugs had larger particle sizes than RA and TS drugs [34]. It provides evidence that the larger the particles of an active pharmaceutical ingredient, the less soluble it was. Identical drug solubility is only possible in drugs with identical particle sizes. However, in contrast to our study, the scientists used laser diffraction analysis in the abovementioned work [34] to identify differences in the API particle size distribution. Like the quantitative optical microscopy we used in this work, laser diffraction analysis is a direct method of studying the size distribution of nimesulide API particles. But this method requires expensive specialized equipment [25], highly qualified and specially trained personnel, complex preparation for the final analytical process of the test, so it is not rapid and easy to perform. Based on this, in our opinion, laser diffraction analysis will be difficult to use as a "generally accessible" method of pilot detection of abnormal lots of generic drugs nimesulide on the market, in contrast to the proposed method of pharmacopoeial quantitative optical microscopy.

If we take into account other studies different from our study, it is in the study of Bojnanska E. et al. reported almost all critical parameters that affect surrogate bioavailability *in vitro* (dissolution rate in the pharmaceutical Dissolution test) [35]. Namely: particle size distribution, specific surface area of particles, Zeta potential and water content, except for polymorphism (if any). The authors concluded that zeta potential and the particle size distribution are the most important parameters. Zeta potential determines the tendency of dispersion particles to form agglomerates and thus prevent the formation of a true solution. The lower the zeta potential is, the higher the tendency to form such agglomerates. And since the absorption of API from the GIT can occur only if the API is in the form of a true solution, then the higher the Zeta Potential is, the more favorable the conditions for dissolution. Zeta Potential is determined through complex electrokinetic studies and requires special expensive equipment [36, 37]. In addition, in our opinion, Zeta Potential is parameter mostly derived from the particle size for a given API with all other parameters being equal, i.e. when comparing the same crystal form. Determining Zeta Potential requires special expensive equipment. Therefore, the size distribution of API particles is the remaining main parameter, which can be determined by various direct methods, one of which is optical microscopy method, the simplest and "generally accessible" for most countries, including Ukraine [21, 22].

In contrast to our direct method for studying the particle size distribution of nimesulide API using optical microscopy, Santos B.W.L. et al. carried out the identification of differences in the bioavailability of generic drugs from the original using an indirect method - the Dissolution test [38]. Through this test, the researchers found that two of the three generic investigational drugs were statistically different in dissolution efficiency from the original drug and were characterized by rapid drug release (more than 85% within 15 min after analysis). This testified to the discrepancy between the studied generic drugs and the original one and ruled out their interchangeability. However, the test methodology and the Dissolution test require a highly specialized laboratory, special equipment, and not only to perform the test itself, but also to carry out the final analytical operation – spectrophotometry or high-performance liquid chromatography with mass spectrometric detection (HPLC-MS). Based on the mentioned above, using the Dissolution test requires considerable time and material costs. This test, in our opinion, is more rational to use after confirmation of the need for its use by means of a pilot comparative analysis of generic and original granules of nimesulide using optical microscopy.

Increased drug solubility in case of smaller API particle size is confirmed in another study [39]. Studying the nanocrystal structure of a nimesulide drug, the scientists found that better solubility of BCS Class II drugs (including nimesulide) in water can be achieved by reducing the particle size using nanotechnologies. It is also confirmed by R. Rascioni et al.’s study [40], which found a relation between the reduction of the nimesulide particle size and the increased dissolution rate.

However, Wei W. et al. in the study of investigation the ways to increase the dissolution and bioavailability of nimesulide, the researchers found that mechanical grinding of pure nimesulide API did not sufficiently improve the solubility in water. Manufacturing the solid dispersions of nimesulide with additional ingredients and subsequent mechanical treatment in a roll mill allowed to achieve an increase in dissolution of 2.6–107 times, depending on the grinding time [41]. However, other researchers have proven a significant effect of the API particle size on the bioavailability of drugs [13]. Biljana Tubi et al. in their study to compare the dissolution of 4 different solid nimesulide drugs in the medium with pH 7.5 found that micronized API particles allowed to achieve the maximum dissolution profile of the drug with surfactants in the composition additional substances for 15 min and drug dissolution within 60 min without surfactants. The researchers found that the API particles size had a greater effect on the dissolution profile than the presence or absence of surfactants in the composition.

Based on the above scientific findings, the solubility of API particles considerably depends on the geometry of the particles. Small particle sizes increase the solubility and the dissolution rate of the API. On the contrary, large particles decrease API solubility, inhibit API dissolution and affect drug bioavailability. Taking into account the above findings and the crystallography of our study, we have obtained results that demonstrate a reliable difference in bioavailability and bioequivalence of generic NB, NC and ND drugs as compared to the innovative NA drug judging by the reliable differences (р<0.05) in the distribution of their particles by size and quantity. The difference between the generic drugs and the original drug may be due to the non-compliance with and violation of the manufacturing conditions or to the use of outdated technologies. Thus 81–240 μm particles were discovered in the NC drug, and 81–400 μm were discovered in the ND drug. The original NA drug did not contain particles of these sizes. Large particles lead to incomplete dissolution of the drug in the GIT, which reduces clinical effectiveness and increases the risk of side effects [42]. In addition, the failure to achieve a clinical effect due to partial dissolution encourages patients to take higher doses of the drug, which can lead to undesirable side effects, including complications from the GIT [41]. Thus, it can be assumed that the bioavailability of NC and ND samples are lower as compared to NA.

NB almost does not contain particles larger than 81 μm, but the number of particles with the minimum size 31–50 μm is reliably different from the amount of such particles in the original NA drug (χ^2^ – 15.15, р<0.01). According to the method of quantitative optical microscopy that we used, the entire sample area was analyzed for nimesulide API particles exceeding 30 μm. Therefore, the analysis of nimesulide particles smaller than the specified 30 μm was not available in the results of this study and for further discussion.

Based on the results of pilot surveys and laboratory tests and taking into account crystallography and the above scientific publications, a conclusion can be made. A reliable increase (р<0.001) in the number of large 121–400 μm particles in the NC and ND drugs may increase the time of dissolution and release of the API in the GIT and lead to a shift in the onset of drug action and API release in distal GIT sections. Conversely, a multifold increase in the number of small particles in the NB drug (р<0.01) may cause extra fast dissolution and API release at the beginning of the digestive tract, in the stomach. The mismatch between the size and distribution of particles in generic nimesulide drugs as compared to the original drug may explain the GIT-associated side effects related to the administration of generic NSAIDs, as noted by the respondents. The results obtained provide evidence of possible differences in the bioavailability and bioequivalence of generic NB, NC and ND drugs as compared to the original NA drug and justify the need for further in-depth research aimed to prevent complications when administering these drugs.

## 5 Conclusion

It is found as a result of pilot anonymous surveying of 147 dentists that specialists note a practical difference in the effects of the administration of original and generic nonsteroidal anti-inflammatory drugs. In the surveys, dentists pointed to a higher percentage of side effects and lower clinical efficacy when administering generic nonsteroidal anti-inflammatory drugs.

Using the proposed technique of optical microscopy for pilot comparative analysis of generic and original nimesulide granules, anomalous lots of three commercially available generic drugs, NB, NC and ND, were identified. The significant difference in the size and distribution of particles in the generic drugs as compared to the original NA suggests possible differences in bioavailability and bioequivalence.

Using the optical microscopy for pilot comparative analysis of particle sizes and distribution in commercially available generic drugs can rapidly and with minimal costs detect anomalous lots of these drugs on the market, predict the loss of clinical effectiveness and prevent potential complications when the drugs are administered for the prevention of inflammation in the dental practice.

In case of pilot detection of anomalous lots of generic drugs on the market by using the optical microscopy to determine the further question about prohibition their use and withdrawal from the market requires additional costly laboratory tests of bioavailability and bioequivalence of these lots to the original drugs.

## Declarations

### Author contribution statement

Ostanina Natalia; Leonenko Pavlo; Levin Mykhaylo: Conceived and designed the experiments; Analyzed and interpreted the data; Wrote the paper.

Kokoieva Yuliia; Leonenko Halyna: Analyzed and interpreted the data; Wrote the paper.

Gumeniuk Oleksii: Performed the experiments; Analyzed and interpreted the data; Contributed reagents, materials, analysis tools or data.

Doroshenko Olena: Analyzed and interpreted the data; Contributed reagents, materials, analysis tools or data.

Nikolaieva Yana: Performed the experiments; Contributed reagents, materials, analysis tools or data.

### Funding statement

The work was supported by the state-funded scientific program “Development of the methodology for choosing generic drugs to provide the Ukrainian population with high-quality drugs”, topic code АМН.14.18 and the state funding of the PhD thesis “Personalized approach to direct prosthetics on dental implants”, state registration number 0117U006528.

### Data availability statement

Data will be made available on request.

### Declaration of interests statement

The authors declare no conflict of interest.

### Additional information

No additional information is available for this paper.
